# Damaged Mesenchymal Cells Dampen the Inflammatory Response of Macrophages and the Formation of Osteoclasts

**DOI:** 10.3390/jcm11144061

**Published:** 2022-07-14

**Authors:** Layla Panahipour, Azarakhsh Oladzad Abbasabadi, Viktoria Kaiser, Mariane Beatriz Sordi, Zahra Kargarpour, Reinhard Gruber

**Affiliations:** 1 Department of Oral Biology, University Clinic of Dentistry, Medical University of Vienna, 1090 Vienna, Austria; layla.panahipour@meduniwien.ac.at (L.P.); az.azar66@gmail.com (A.O.A.); n01505235@students.meduniwien.ac.at (V.K.); mariane.sordi@kcl.ac.uk (M.B.S.); zahra.kargarpooresfahani@meduniwien.ac.at (Z.K.); 2 Department of Dentistry, Federal University of Santa Catarina, Florianopolis 88040-900, Brazil; 3 Department of Periodontology, School of Dental Medicine, University of Bern, 3010 Bern, Switzerland; 4 Austrian Cluster for Tissue Regeneration, 1200 Vienna, Austria

**Keywords:** mesenchymal cell lysates, macrophage, osteoclast, inflammation

## Abstract

Damage to mesenchymal cells occurs by dental implant drills as a consequence of shear forces and heat generation. However, how the damaged mesenchymal cells can affect the polarization of macrophages and their differentiation into osteoclastogenesis is not fully understood. To simulate cell damage, we exposed suspended ST2 murine bone marrow stromal cells to freeze/thawing or sonication cycles, followed by centrifugation. We then evaluated the lysates for their capacity to modulate lipopolysaccharide-induced macrophage polarization and RANKL-MCSF-TGF-β-induced osteoclastogenesis. We report that lysates of ST2, particularly when sonicated, greatly diminished the expression of inflammatory IL6 and COX2 as well as moderately increased arginase 1 in primary macrophages. That was confirmed by lysates obtained from the osteocytic cell line IDG-SW3. Moreover, the ST2 lysate lowered the phosphorylation of p65 and p38 as well as the nuclear translocation of p65. We further show herein that lysates of damaged ST2 reduced the formation of osteoclast-like cells characterized by their multinuclearity and the expression of tartrate-resistant phosphatase and cathepsin K. Taken together, our data suggest that thermal and mechanical damage of mesenchymal cells causes the release of as-yet-to-be-defined molecules that dampen an inflammatory response and the formation of osteoclasts in vitro.

## 1. Introduction

Implant dentistry is based on two pillars: the primary mechanical stability that later on turns into the secondary biological stability [1]. The primary stability of dental implants is achieved by drilling and screwing an implant into bone, aiming to achieve firm frication. Drilling, however, is an invasive procedure generating mechanical damage, particularly in the area immediately adjacent to the implant that, by its insertion, also causes tissue damage, especially when self-cutting and conical in shape [2,3]. Consequently, bone experiences thermal and mechanical stresses. Damaged bone signals the need to be replaced by new bone [4], and it is the new bone that initiates the formation of a secondary biological stability [5]. New bone connects the pristine bone with the surface of the implant, providing a biological anchorage of the implant in the alveolar bone.

Damaged bone initiates a catabolic process where macrophages are activated and receive signals that incite their differentiation towards bone-resorbing osteoclasts [4]. Increasing evidence suggests that these signals are potentially derived from mesenchymal cells of the bone marrow [6], in particular those spanning the bone, namely the osteocytes [7,8]. Dying osteocytes release ligands that support the RANKL-induced osteoclastogenesis, suggesting a direct link between osteocyte necrosis and bone resorption [9,10]. In vitro, scratching or serum-starvation of the osteocyte-like cells MLO-Y4 support osteoclastogenesis in bone marrow culture [11,12]. In addition, necrotic IDG-SW3 osteocytic cells support in vitro osteoclastogenesis [13]. This information has culminated in the design of drills and implants overall aiming to reduce bone damage and thus maintain the integrity of osteocytes [14,15]. It is, however, not only the bone that is deteriorated; it is the integrity of the overall tissue that is affected. Necrotic disruption of the bone marrow and its mesenchymal cells cause the release of the cellular content into the early peri-implant space where macrophages and, later in the downstream, also osteoclasts are present.

All injured cells release danger signals, also called alarmins that alert the host to cell death [16,17]. It can thus be hypothesized that macrophages respond to the necrotic disrupted bone marrow mesenchymal cells. The crosstalk between mesenchymal cells and macrophages in the development of disease and maintenance of homeostasis of inflammatory microenvironments became a research focus [18,19]. However, evidence supporting a role of damaged mesenchymal cell to modulate osteoclastogenesis and macrophage polarization [20] is limited. Thus, damaged mesenchymal cells can potentially modulate the local innate immunity, a process that is responsible for microbial defense and modulation of the cellular events of tissue healing. This accumulating knowledge has prompted us to investing how bone-marrow-derived mesenchymal cells that experienced mechanical damage affect the classical macrophage responses, which are the lipopolysaccharides (LPS)-induced expression of inflammatory mediators and their ability to become osteoclasts when grown in the presence of RANKL-MCSF-TGF-β1.

The present in vitro study takes advantage of the established cell line ST2 originating from murine bone marrow originally used to stimulate osteoclastogenesis [21]. Moreover, we implemented the osteocytic cell line IDG-SW3 in our setting [22]. Cell damage occurring during drilling and implant placement was simulated by sonication and freeze/thawing cycles. The respective cell lysates were then used to modulate the LPS response of RAW 264.7 macrophages and primary macrophages from murine bone marrow. Likewise, the lysates of ST2 cells were studied for their impact on the differentiation of murine bone marrow cells towards the osteoclastogenic lineage. Based on this in vitro setting, we aimed to study how damaged mesenchymal cells in bone marrow affect the polarization of macrophages and their differentiation into osteoclasts. 

## 2. Methods

### 2.1. Mesenchymal Cell Lysate

The ST2 mesenchymal stromal cell line was originally isolated from mouse bone marrow (Riken Cell Bank, Tsukuba, Japan). The cells were expanded in growth Dulbecco’s modified Eagle’s medium (DMEM, Sigma-Aldrich, St. Louis, MO, USA), 10% fetal calf serum (Bio&Sell GmbH, Nuremberg, Germany), and 1% antibiotics (Sigma Aldrich). IDG-SW3 osteocytic cell line (Kerafast, Inc., Boston, MA, USA) was expanded as recommended [22]. To prepare the lysates, ST2 and IDG-SW3 cells were suspended in serum-free DMEM for macrophage cultures. For osteoclast culture, ST2 cells were suspended in alpha Minimum Essential Medium (αMEM) supplemented with 10% fetal bovine serum and antibiotics (all from Invitrogen, Grand Island, NY, USA). The cell suspensions were subjected to the following treatments: (i) three cycles of freeze/thawing for 8 min at −80 °C and room temperature and (ii) three cycles of sonication for 15 s (Sonoplus; Bandelin electronic GmbH & Co., KG; Berlin, Germany). The lysates underwent centrifugation at 2600 RCF for 5 min (5420; Eppendorf SE, Hamburg, Germany). All supernatants, now considered cell lysates, were always freshly prepared for experimentation.

### 2.2. Bone-Marrow-Derived Macrophages and RAW 264.7 Cells

BALB/c mice with ages of 6 to 8 weeks were bought from Animal Research Laboratories, Himberg, Austria. Bone marrow cells were derived from the femora and tibiae as reported earlier [23]. Bone marrow cells were cultured at 1 × 10^6^ cells/cm^2^ in 24-well plates and left to grow for 5 days in DMEM containing 10% fetal bovine serum, antibiotics, and 20 ng/mL macrophage colony-stimulating factor (M-CSF; ProSpec, Ness-Ziona, Israel). RAW 264.7 macrophage-like cells were expanded in growth medium only and seeded at 1 × 10^6^ cells/cm^2^ into 24-well plates. Bone marrow macrophages and RAW 264.7 were treated by lipopolysaccharide from *Escherichia coli 0111: B41* (LPS; Sigma Aldrich, St. Louis, MO, USA) at 100 ng/mL in the presence and absence of ST2 and IDG-SW3 lysates overnight.

### 2.3. Bone-Marrow-Derived Osteoclasts

Bone marrow cells were cultured at 1 × 10^6^ cells/cm^2^ in 24-well plates and continued to grow for 5 days in αMEM with 10% fetal calf serum and antibiotics. Recombinant RANKL at 30 ng/mL, M-CSF at 20 ng/mL, and TGF-β1 at 10 ng/mL (all from ProSpec, Ness-Ziona, Israel) were applied to the culture medium to induce osteoclastogenesis in the presence and absence of ST2 lysates. Six days later, histochemical staining for TRAP was carried out (Sigma Aldrich, St. Louis, MO, USA). Images were taken by a light microscope with 20× magnification (Oxion fluorescence, Euromex, Arnheim, The Netherlands). 

### 2.4. Reverse Transcription Quantitative Real-Time PCR (RT-qPCR) and Immunoassay

Total RNA was extracted using the ExtractMe total RNA kit (Blirt S.A., Gdańsk, Poland). Next, complementary DNA (cDNA) was synthesized through reverse transcription of the total RNA (LabQ, Labconsulting, Vienna, Austria) Polymerase chain reaction was performed (LabQ, Labconsulting, Vienna, Austria) on a CFX Connect™ Real-Time PCR Detection System (Bio-Rad Laboratories, Hercules, CA, USA). Sequences of the primers were IL6-F: GCTACCAAACTGGATATAATCAGGA, IL6-R: CCAGGTAGCTATGG-TACTCCAGAA; COX2-F: CAGACAACATAAAACTGCGCCTT, COX2-R: GATACACCTCTCCACCAATGACC; IL1-F: TTGGTTAAATGACCTGCAACA, IL1-R: GAGCGCTCACGAACAGTTG; GAPDH-F: AACTTTGGCATTGTGGAAGG, GAPDH-R: GGATGCAGGGATGATGTTCT; ARG1-F: GAATCTGCATGGGCAACC, ARG1-R: GAATCCTGGTACATCTGGGAAC; CTSK-F: TGTATAACGCCACGGCAAA, CTSK-R: GGTTCACATTATCACGGTCACA; TRAP-F: AAGCGCAAACGGTAGTAAGG, TRAP-R: CGTCTCTGCACAGATTGCAT. The amount of each specific mRNA was normalized to the housekeeping gene GAPDH by the ^ΔΔ^Ct method. RT-qPCR data are depicted compared to the unstimulated control, which was assumed as 1.0 in all the analyses. Supernatants of each well representing an independent sample were measured for IL6 secretion by immunoassay (R&D Systems, Minneapolis, MN, USA). 

### 2.5. Immunofluorescence Analysis

The immunofluorescent examination of nuclear translocation of NFκB-p65 was implemented in RAW 264.7 cells seeded into Millicell^®^ EZ slides (Merck KGaA, Darmstadt, Germany) at 1 × 10^6^ cells/cm^2^. Cells were first overnight serum-deprived and next pretreated with ST2 and IDG-SW3 lysates. Afterwards, the cells were stimulated with 100 ng/mL LPS for another 30 min. The cells were fixed using 4% paraformaldehyde and blocked with 1% bovine serum albumin (BSA, Sigma Aldrich, St. Louis, MO, USA). The anti-NF-κB p65 antibody (IgG, 1:800, Cell Signaling Technology, CST, Cambridge, UK, #8242) was added to the cells at 4 °C overnight followed by the goat anti-rabbit Alexa 488 (1:1000, CST, #4412) and Fluoromount-GTM (Invitrogen, Carlsbad, CA, USA). Images were taken using the fluorescent microscope with a dual excitation filter block DAPI-FITC (Echorevolve Fluorescence microscope, San Diego, CA, USA)

### 2.6. Western Blot

RAW 264.7 cells at 1 × 10^6^ cells/cm^2^ were seeded into 12-well plates. The next day, cells were treated with ST2 lysates for 30 min and then exposed to LPS for another 30 min. Cell lysates were prepared using SDS buffer containing protease and phosphatase inhibitors (cOmplete ULTRA Tablets and PhosSTOP; Roche, Mannheim, Germany). Cell lysates were subjected to SDS-PAGE and transferred onto PVDF membranes (Roche Diagnostics, Mannheim, Germany). Membranes were blocked and the first antibodies bound: NFκB-p65 antibodies (IgG, 1:1000, CST, #8242) and phospho-NFκB-p65 antibody (IgG, 1:1000, CST, #3033), p38 (IgG, 1:1000, SC-#535) and phospho-p38 antibody (IgG, 1:1000, SC, #4511), which were identified with the second antibody labelled with HRP anti-rabbit (IgG, 1:10,000, CST, #7074). Following incubation with Clarity Western ECL Substrate (Bio-Rad Laboratories, Inc., Hercules, CA, USA), chemiluminescence signals were envisioned with the ChemiDoc imaging system (Bio-Rad Laboratories). 

### 2.7. Statistical Analysis

The experiments were repeated at least four times. Every single data point belongs to an individual experiment. Statistical analyses were based on one-way ANOVA and Friedman test. Analyses were performed using Prism v8 (GraphPad Software, La Jolla, CA, USA). Significance was set at *p* < 0.05.

## 3. Results

### 3.1. ST2 Lysates Reduce LPS-Induced IL6 and COX2 in RAW 264.7 Cells

**Figure 1:**
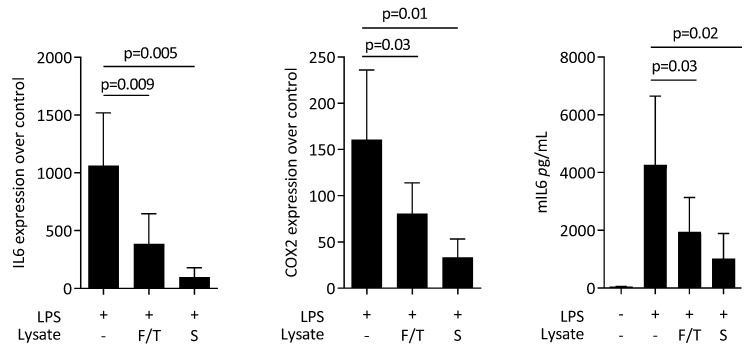
ST2 lysates reduce lipopolysaccharides (LPS)-induced M1 polarization of RAW 264.7 macrophages. RAW 264.7 macrophages were exposed to ST2 lysates prepared by freeze/thawing (F/T) and sonication (S) with LPS. Data show the x-fold changes of IL6 and COX2 gene expression and the release of IL6 protein into the supernatant. The expression changes were normalized to an unstimulated control. The bars represent the mean and standard deviation of five independent experiments. Statistical analyses were based on RM one-way ANOVA.

First, to rule out any cytotoxic effects of ST2 lysates, we performed an MTT assay. When RAW 264.7 macrophages were exposed to the murine bone marrow stromal cell lysates, no changes compared to a serum-free medium control were noticed regarding cytotoxicity (data not shown). We then assessed the impact of ST2 lysates on LPS-induced polarization of RAW 264.7 macrophages towards a pro-inflammatory M1 phenotype. RT-PCR analyses of IL6 and COX2 and immunoassay of IL6 protein were conducted. LPS caused the expected increased expression of IL6 and COX2 at the transcriptional level and of IL6 at the protein level. This LPS-response was significantly lowered by the presence of ST2 lysates, particularly when prepared by sonication (Figure 1).

### 3.2. ST2 Lysates Moderately Reduce LPS-Induced NFκB and p38 Signaling in RAW 264.7 Cells

**Figure 2:**
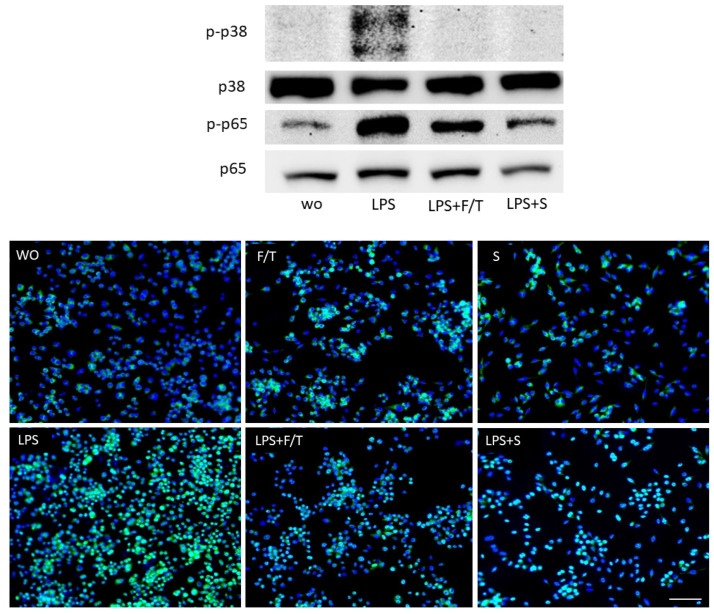
ST2 lysates reduce LPS-induced NFκB and p38 signaling in RAW 264.7 cells. RAW 264.7 macrophages were exposed to ST2 lysates prepared by freeze/thawing (F/T) and sonication (S) with and without LPS, aiming to induce the phosphorylation and nuclear translocation of p65, together with phosphorylation of p38. Western blot analysis shows the chemiluminescence signals obtained with the phosphor-p65 and p65 and phosphor-p38 and p38 antibodies. Immunostaining revealed the green fluorescence signals obtained with the p65 antibody. Nuclear staining with DAPI appears blue. Scale bar indicates 100 µm and represents all pictures.

To further assess how ST2 lysates modulate the inflammatory response in RAW 264.7 cells, activation of NFκB-p65 and p38 signaling at the level of phosphorylation and NFκB-p65 nuclear translocation were performed by Western blot and immunostaining, respectively. In line with the decreased expression of inflammatory markers, ST2 lysates prepared by sonication and by freeze/thawing to a lesser extent, noticeably reduced the p38 and NFκB-p65 phosphorylation levels (Figure 2). Immunostaining revealed a reduction of p65 nuclear translocation in the presence of ST2 lysates (Figure 2). These findings suggest that ST2 lysates are capable to reduce the LPS-induced activation of p38 and NFκB signaling in RAW 264.7 cells. 

### 3.3. ST2 Lysates Reduce LPS-Induced M1 Polarization in Primary Macrophages

**Figure 3:**
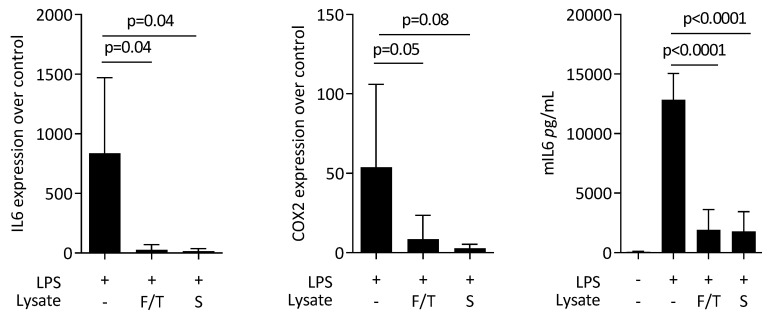
ST2 lysates reduce LPS-induced M1 polarization in primary macrophages. Primary bone-marrow-derived macrophages were exposed to ST2 lysates prepared by freeze/thawing (F/T) and sonication (S) with LPS. Data show the x-fold changes of IL6 and COX2 gene expression as well as the release of IL6 protein into the supernatant. The expression changes were normalized to an unstimulated control. The bars represent the mean and standard deviation of five independent experiments. Statistical analyses were based on RM one-way ANOVA.

**Figure 4:**
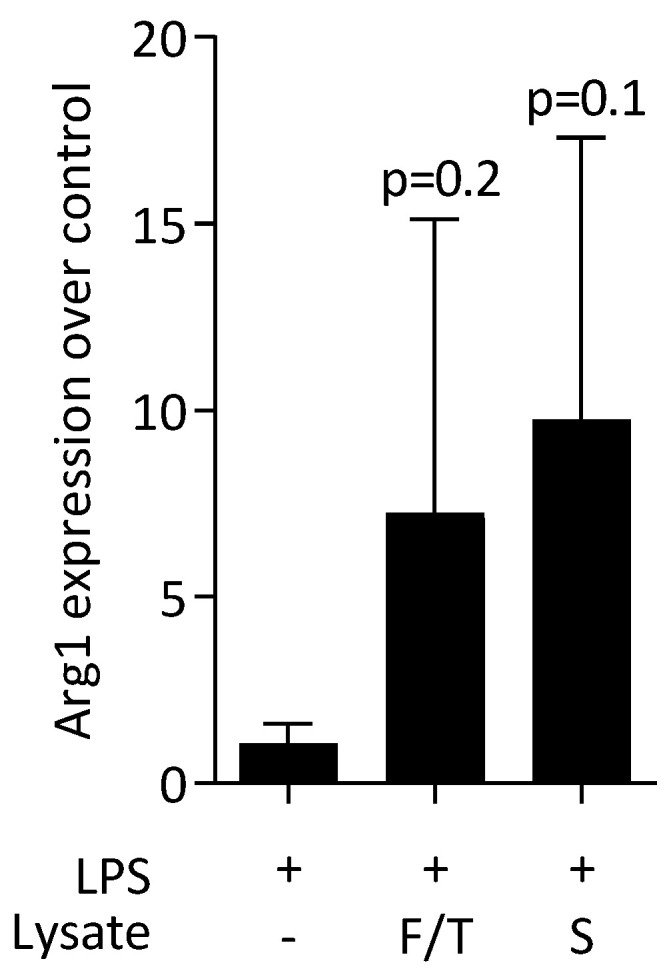
ST2 lysates increase M2 polarization in primary macrophages. Primary bone-marrow-derived macrophages were exposed to ST2 lysates prepared by freeze/thawing (F/T) and sonication (S). The expression changes of arginase-1 (Arg1) were normalized to an unstimulated control. The bars represent the mean and standard deviation of four independent experiments. Statistical analysis was based on Friedman test.

Next, we evaluated the effect of ST2 lysates on LPS-induced polarization of primary macrophages towards a pro-inflammatory phenotype. LPS again caused a robust increase in the expression of IL6 and COX2 at transcriptional level and for IL6 on the protein level (Figure 3). Consistent to what was observed with RAW 264.7 cells, ST2 lysates significantly reduced the inflammatory response of the primary macrophages to LPS. Moreover, there was an increase of the M2 marker arginase-1 (Figure 4).

### 3.4. IDG-SW3 Lysates Reduce LPS-Induced M1 Polarization in Primary Macrophages

**Figure 5:**
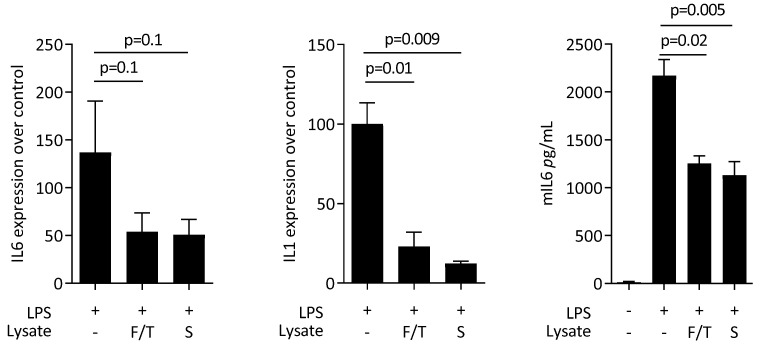
IDG-SW3 lysates reduce LPS-induced M1 polarization in primary macrophages. Primary bone-marrow-derived macrophages were exposed to IDG-SW3 lysates prepared by freeze/thawing (F/T) and sonication (S) with LPS. Data show the expression of IL6 and IL1 and the release of IL6 protein in the supernatant. The bars represent the mean and standard deviation of three independent experiments. Statistical analyses were based on RM one-way ANOVA.

To confirm that the observed effects are not restricted to one particular mesenchymal cell line, we prepared lysates from IDG-SW3 cells, an osteocytic cell line [22]. In line with what we observed with ST2 cells, the IDG-SW3 lysates significantly diminished the LPS-induced expression of IL6 and IL1 in primary macrophages (Figure 5).

### 3.5. ST2 Lysates Diminish Osteoclastogenesis of Bone Marrow Cells

**Figure 6:**
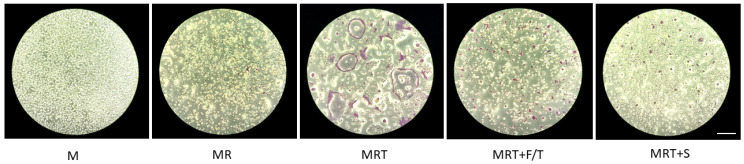
ST2 lysates reduce osteoclast differentiation induced by RANKL, MCSF, and TGF-β. Primary bone marrow cells were grown in the presence and absence of ST2 lysates prepared by freeze/thawing (F/T) and sonication (S) to modify osteoclastogenesis induced by of RANKL (R), MCSF (M), and TGF-β (T). Histochemical staining represents the activity of the dark-red staining TRAP+ multinucleated osteoclasts. Note that the presence of ST2 lysates diminished the number but also lowered the size of the remaining TRAP-positive cells. Scale bar indicates 100 µm and is representative for all pictures.

**Figure 7:**
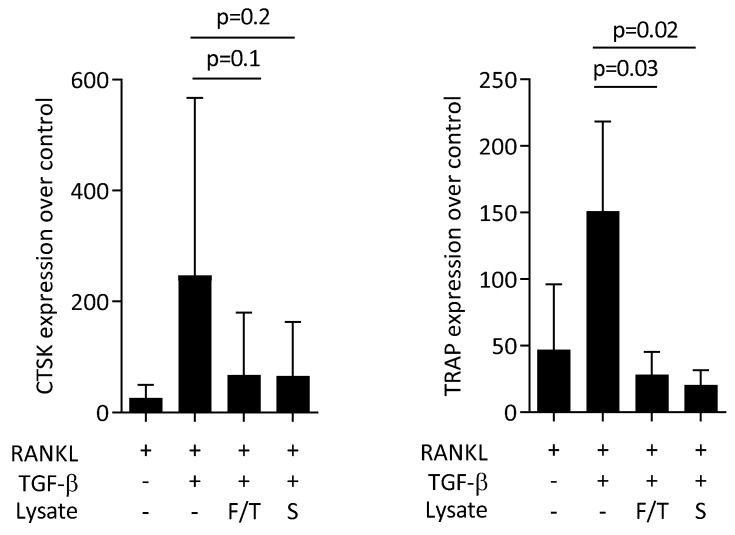
ST2 lysates reduce osteoclastogenesis in primary bone marrow cultures. Primary bone-marrow-derived macrophages were grown in the presence of RANKL-MCSF-TGF-β with and without ST2 lysates prepared by freeze/thawing (F/T) and sonication (S). Data represent the x-fold changes in gene expression compared to an MCSF control. The bars represent the mean and standard deviation of four independent experiments. Statistical analyses were based on RM one-way ANOVA.

Finally, we questioned if ST2 lysates would affect the process of osteoclastogenesis. To this aim, we grew primary bone marrow cells in the presence of RANKL-MCSF-TGF-β1 to push osteoclastogenesis with and without the ST2 cell lysates. Histochemical staining revealed that ST2 lysates greatly reduced the formation of large multinucleated cells staining positive for the osteoclast marker TRAP (Figure 6). Gene expression analysis further confirmed that the presence of the ST2 lysates diminished the expression of cathepsin K and TRAP (Figure 7). Thus, lysates of ST2 cells can decrease the process of in vitro osteoclastogenesis. 

## 4. Discussion

This research was inspired by the concept that cell damage that occurs as a consequence of implant placement into the alveolar bone contributes to the early healing phase where macrophages and osteoclast come into play [2,3]. Drilling and insertion of dental implants cause harm to the bone and the respective bone marrow, affecting its mesenchymal cells [5]. Even though this speculation remains at the level of a hypothesis, it can be assumed that local cells of the mesenchymal lineage, including those of the bone marrow and osteocytes embedded in the bone matrix, are damaged [2,3]. Consequently, the content of the cytoplasm and fragments of the membrane are released into the local environment, possibly modulating the early healing phase. Having this picture in mind, we performed a proof-of-concept study using lysates from murine bone-marrow-derived mesenchymal cell line (ST2) and osteocytic cell line (IDG-SW3) subjected to sonication and freeze/thawing cycles. Our aim was thus to investigate how damaged mesenchymal cells affect macrophage polarization and their differentiation towards osteoclasts. The two main findings were that the cell lysates reduced the LPS-induced inflammatory response of macrophages and dampened the RANKL-MCSF-TGF-β-induced osteoclastogenesis of bone marrow cells. These findings are important because they show that damaged cells of the mesenchymal lineage do not necessarily drive a catabolic process but may lower inflammatory osteolysis. 

If we relate the findings to those of others, we can refer to the work showing that the supernatant of necrotic splenocytes and serum-starved necrotic osteocytic cell line IDG-SW3 enhanced osteoclastogenesis in vitro [13]. Moreover, scratching or serum-starvation of the osteocyte-like cells MLO-Y4 support osteoclastogenesis in bone marrow culture [11,12]. These observations are in contrast to our findings, and the ST2 cells also originally served as a source of RANKL to push osteoclastogenesis in cocultures [21]. However, we tried to simulate a mechanical injury by sonication and freeze/thawing of ST2 cells, while others provoked osteocyte necrosis by the lack of nutrition [11,13]. Moreover, in contrast to our findings are the observations based on soluble mediators in erythrocytes that enhanced monocyte IL1β inflammation [24], and in general, signals released by damaged cells are overall proinflammatory in nature [16,17]. Thus, the consensus is that soluble mediators that are naturally released exert a pro-inflammatory activity on macrophages and support the process of osteoclastogenesis. Nevertheless, we cannot directly compare the robust anti-inflammatory and anti-osteoclastogenic effects we observed with sonicated and freeze/thawing of murine mesenchymal cells with other studies using cell culture supernatants.

The clinical relevance of our findings remains at the level of a speculation considering that the early stages of osseointegration of dental implants are initiated by an inflammatory reaction and osteoclastogenesis [5]. However, this simplified view needs refinement. Our in vitro settings represent an extreme situation where large numbers of bone marrow mesenchymal cells are disrupted, while in vivo, only a limited number of cells is affected by mechanical damage. Hence, in vivo cell damage is limited to the area immediately adjacent site of implant insertion [2]. Thus, the possible implication of damaged cell to control local inflammation and bone regeneration is limited. It is particularly the small area next to the implant that has an impact on the early stage of osseointegration, and thus, it should not be ruled out that the damaged cells target macrophages and modulate osteoclastogenesis in vivo. At the level of our research, it is hard to draw conclusions regarding how a surgeon or specialist in implant placement could follow these concepts and apply to his or her own daily practice. Nevertheless, our data provide the scientific basis of how cell damage affects local inflammation and bone resorption, with implant dentistry used as a possible scenario. 

This pilot study has limitations that need to be considered. Even though our research was inspired by a clinical scenario, it does not necessarily reflect the complex in vivo situation. For instance, we do not know to which extent the ST2 and IDG-SW3 cells represent the stromal cells and osteocytes in the alveolar bone even though both cell lines are established with in vitro tools and thus ideal for proof-of-principle studies [6,22]. Then, we cannot simulate what happens to cells in vivo upon drilling and implant insertion; sonication and freeze/thawing are maybe not ideal settings in this respect. Nevertheless, sonication and freeze/thawing allow a controlled damage that included the disruption of the membrane and the release of the cytoplasmatic content into the cell lysate. Additionally, it is unknown to which extent and if at all the mesenchymal cells of the alveolar bone marrow are damaged upon implant insertion. Thus, the present work is based on a clinical assumption, but nevertheless, our findings can be generalized to the principles of necrotic bone marrow mesenchymal cell. Other limitations are that LPS does not necessarily represent a situation of how macrophages are activated at a defect site. Moreover, and even though the traditional murine bone marrow osteoclastogenesis model is widely used [6], it remains an in vitro bioassay, and findings should be interpreted carefully with respect to the in vivo implication. 

Future research should consider the translation of the murine setting towards using cells of human origin. Furthermore, we need to understand to which extent local cells are damaged by drilling and implant insertion, similar to what has been shown for apoptotic osteocytes [13]. The challenge here is that necrotic cells have no markers, so potentially, the impact of how cells undergoing necroptosis and pyroptosis should be evaluated for their potential to modulate osseointegration. Our research can be extended towards the omics technology because IL6, IL1, and COX2 are indicator cytokines and enzymes representing a broad and complex cellular response. It cannot be ruled out that ST2 and IDG-SW3 lysates even provoke the release of molecules that actively support regeneration, including molecules pushing angiogenesis or the antimicrobial defense by macrophages. Moreover, we need to decipher the molecular mechanisms by which ST2 and IDG-SW3 cell lysates cause the anti-inflammatory and anti-osteoclastic activities. Finally, future research will reveal to which extent the clinical protocols, including the drilling and rinsing, implant insertion, the anatomy, the material properties, as well as the surface design of dental implants, affects local cell damage and potentially the early cellular events of osseointegration. This proof-of-principle study should consequently be considered as a primer for future research. 

## 5. Conclusions

Taken together, the data we provide here suggest that lysates of a bone marrow stromal cell line ST2 and osteocytic cell line IDG-SW3 are able to lower the LPS-induced activation of macrophages and that ST2 lysates reduce the RANKL-MCSF-TGF-β-induced osteoclastogenesis in a bone-marrow-based bioassay. This pilot study paves the way for future research to increase our knowledge of how damaged cells modulate the early stages of osseointegration.

## Data Availability

The original contributions presented in the study are included in the article. Further inquiries can be directed to the corresponding author.
